# Progress Toward Hepatitis B Control and Elimination of Mother-to-Child Transmission of Hepatitis B Virus — Western Pacific Region, 2005–2017

**DOI:** 10.15585/mmwr.mm6808a2

**Published:** 2019-03-01

**Authors:** Joseph Woodring, Roberta Pastore, Anne Brink, Naoko Ishikawa, Yoshihiro Takashima, Rania A. Tohme

**Affiliations:** ^1^Western Pacific Regional Office, Expanded Programme on Immunization, World Health Organization, Manila, Philippines; ^2^Western Pacific Regional Office, HIV, Hepatitis and Sexually Transmitted Infections, World Health Organization, Manila, Philippines; ^3^Global Immunization Division, Center for Global Health, CDC.

Hepatitis B vaccine (HepB), which has been available since 1982, provides lifelong protection against hepatitis B virus (HBV) infection and the associated 20%–30% increased lifetime risk for developing cirrhosis or hepatocellular carcinoma among >95% of vaccine recipients (*1*). Before HepB introduction into national childhood immunization schedules, the estimated hepatitis B surface antigen (HBsAg) prevalence in the World Health Organization (WHO) Western Pacific Region (WPR)* was >8% in 1990 (*2*). In 2005, the WPR was the first WHO region to establish a hepatitis B control goal, with an initial target of reducing HBsAg prevalence to <2% among children aged 5 years by 2012. In 2013, the WPR set more stringent control targets to achieve by 2017, including reducing HBsAg prevalence to <1% in children aged 5 years and increasing national coverage with both timely HepB birth dose (HepB-BD) (defined as administration within 24 hours of birth) and the third HepB dose (HepB3) to ≥95% (*3*). All WPR countries/areas endorsed the Regional Action Plan for Viral Hepatitis in the Western Pacific Region 2016–2020 in 2015 (*4*) and the Regional Framework for the Triple Elimination of Mother-to-Child Transmission of human immunodeficiency virus (HIV), Hepatitis B and Syphilis in Asia and the Pacific 2018–2030 (triple elimination framework) in 2017 (*5*). These regional targets and strategies are aligned with program targets established by the WHO Global Health Sector Strategy on Viral Hepatitis 2016–2021 that aim to reduce HBsAg prevalence among children aged 5 years to ≤1% by 2020 and to ≤0.1% by 2030 (*6*). This report describes progress made to achieve hepatitis B control in the WPR and the steps taken to eliminate mother-to-child transmission (MTCT) of HBV during 2005–2017. During this period, regional timely HepB-BD and HepB3 coverage increased from 63% to 85% and from 76% to 93%, respectively. As of December 2017, 15 (42%) countries/areas achieved ≥95% timely HepB-BD coverage; 18 (50%) reached ≥95% HepB3 coverage; and 19 (53%) countries/areas as well as the region as a whole were verified to have achieved the regional and global target of <1% HBsAg prevalence among children aged 5 years. Continued implementation of proven vaccination strategies will be needed to make further progress toward WPR hepatitis B control targets. In addition to high HepB-BD and HepB3 coverage, enhanced implementation of complementary hepatitis B prevention services through the triple elimination framework, including routine HBsAg testing of pregnant women, timely administration of hepatitis B immunoglobulin to exposed newborns, and antiviral treatment of mothers with high viral loads, will be needed to achieve the global hepatitis B elimination target by 2030.

## Immunization Activities

HepB-BD and HepB3 coverage data are reported annually to WHO and the United Nations Children’s Fund (UNICEF) from 36 of the 37 WPR countries and areas.^†^ WHO and UNICEF estimate vaccination coverage for 27 countries in the region, using annual government-reported survey and administrative data; for the remaining areas and territories, government-reported coverage data are used. By 2005, all countries/areas in the region had introduced at least three HepB doses into national immunization schedules. By 2012, 34 (94%) of 36 countries/areas provided universal HepB-BD (Table 1); since 1987, Japan and New Zealand have provided selective administration of timely HepB-BD to infants born to mothers who are HBsAg-positive or whose HBsAg status is unknown. During 2005–2017, regional HepB-BD coverage increased from 63% to 85%, and HepB3 coverage increased from 76% to 93%. In 2017, 15 (42%) of 36 countries/areas achieved ≥95% timely HepB-BD coverage, and 18 (50%) countries/areas reached ≥95% HepB3 coverage.

**TABLE 1:** Hepatitis B vaccine (HepB) schedule and estimated coverage* with a timely birth dose^†^ and third dose of HepB, by country/area — World Health Organization (WHO) Western Pacific Region, 2005, 2012, and 2017

Country/Area	HepB schedule	Year birth dose introduced	% Coverage					
			2005		2012		2017	
			Timely HepB-BD^†^	HepB3	Timely HepB-BD^†^	HepB3	Timely HepB-BD^†^	HepB3
American Samoa	0, 1 mo, 12 mos	1991	100	80	100 (2011)	77 (2011)	NR	NR
Australia	0, 2 mos, 4 mos, 6 mos	2000	NR	95	NR	91	NR	95
Brunei	0, 2 mos, 4 mos, 6 mos	1988	96	99	95	99	99	99
Cambodia	0, 6 wks, 10 wks, 14 wks	2005	NR	82^§^	68	91	79	93
China	0, 1 mo, 6 mos	1992	86	84	96	99	96	99
Commonwealth of Northern Mariana Islands	0, 6 wks, 4 mos, 6 mos	1988	99	89	98	76	95	71
Cook Islands	0, 6 wks, 3 mos, 5 mos	1989	99	99	97	98	99	99
Federated States of Micronesia	0, 2 mos, 4 mos, 6 mos	1988	93	91	81	82	75	80
Fiji	0, 6 wks, 10 wks, 14 wks	1989	90	99	90	99	90	99
French Polynesia	0, 2 mos, 10 mos	1992	100	97	98 (2014)	99	98	98
Guam	0, 1–2 mos, 6–18 mos	1988	98	88	100	38	80	83
Hong Kong SAR (China)	0, 1 mo, 6 mos	1988	100	95	95	95	95	95
Japan	2 mos, 3 mos, 7 mos	1986^¶^	NA^¶^	NR	NA^¶^	NR	NA^¶^	NR
Kiribati	0, 6 wks, 10 wks, 14 wks	1990	70	50	82	94	89	90
Laos	0, 6 wks, 10 wks, 14 wks	2004**	2^§^	49	NR	79	55	85
Macao SAR (China)	0, 1 mo, 6 mos	1989	100	88	100	94	100	95
Malaysia	0, 1 mo, 6 mos	1989	90	96	91	97	90	98
Marshall Islands	0, 2 mos, 4 mos, 6 mos	1998	99	89	96	80	97	82
Mongolia	0, 2 mos, 3 mos, 4 mos	1991	89	98	97	99	98	99
Nauru	0, 6 wks, 10 wks, 14 wks	NK	98	80	99	79	99	87
New Caledonia	0, 2 mos, 11 mos	NK	99 (2007)	99 (2006)	98	93	98	93
New Zealand	6 wks, 3 mos, 5 mos	1987^¶^	NA^¶^	87	NA^¶^	93	NA^¶^	94
Niue	0, 6 wks, 3 mos, 5 mos	NK	9	85	99	98	84	99
Palau	0, 2 mos, 6 mos	1989	99	98	99	89	99	98
Papua New Guinea	0, 1 mo, 2 mos, 3 mos	2003**	35 (2006)	63	35	68	33	56
Philippines	0, 6 wks, 10 wks, 14 wks	2007	NA	49	39	88	67	88
Republic of Korea	0, 1 mo, 6 mos	1983	98^§^	99	92 (2014)^§^	99	92	98
Samoa	0, 6 wks, 10 wks, 14 wks	NK	52	51	83	82	81	73
Singapore	0, 1 mo, 5 mos	1987	74	96	67	97	91	96
Solomon Islands	0, 6 wks, 10 wks, 14 wks	2005**	80	83	59	99	67	99
Tokelau	0, 6 wks, 10 wks, 14 wks	1990	100	100	56	100	100	100 (2016)
Tonga	0, 6 wks, 10 wks, 14 wks	1988	89	89	84	77	88	81
Tuvalu	0, 6 wks, 10 wks, 14 wks	NK	99	79	99	97	99	96
Vanuatu	0, 6 wks, 10 wks, 14 wks	1989–1990	92 (2006)^§^	61	79^§^	79	75^§^	85
Vietnam	0, 2 mos, 3 mos, 4 mos	2005	62^§^	94	76	97	77	94
Wallis and Futuna	0, 2 mos, 11 mos	2006	N/A	100 (2003)	100	>100^§^	97	>100^§^
**Western Pacific Region**	**―**	**―**	**63**	**76**	**80**	**93**	**85**	**93**
**Global**	**―**	**―**	**23**	**54**	**34**	**80**	**43**	**84**

**Abbreviations:** HepB-BD = birth dose of monovalent hepatitis B vaccine; HepB3 = third dose of hepatitis B containing vaccine; NA = not applicable; NK = not known; NR = not reported; SAR = special autonomous region; UNICEF = United Nations Children’s Fund.

* WHO-UNICEF estimates except for areas and territories (American Samoa, French Polynesia, Guam, Hong Kong, Macao, New Caledonia, Commonwealth of Northern Mariana Islands, Tokelau, Wallis, and Futuna), and where otherwise specified.

^†^ Timely hepatitis B birth-dose is defined as administration of a dose of hepatitis B vaccine within 24 hours of birth.

^§^ WHO-UNICEF estimates not available; reported coverage used instead.

^¶^ Year of introduction of selective birth dose vaccination of newborns of mothers who are HBsAg positive or of unknown HBsAg status.

** Approximate year of birth dose introduction into the routine immunization program.

## HBsAg seroprevalence surveys

Surveillance for acute hepatitis B infection and its sequelae cannot fully capture the population prevalence of HBV infections, because most infants and children remain asymptomatic during acute infection. Nationally representative HBsAg seroprevalence surveys allow countries to assess the prevalence of chronic HBV infection among children born after the introduction of hepatitis B vaccine in the national immunization schedule and track progress toward achievement of regional hepatitis B control targets. In 1990, before HepB was introduced into childhood immunization schedules in most WPR countries/areas, HBsAg seroprevalence among children aged 5 years was estimated to be >8% in 22 (61%) of 36 countries/areas (*2*), a level of chronic infection considered to be highly endemic (*1*). As of December 2017, all countries/areas in the WPR had completed serosurveys except for Nauru and New Caledonia; seven countries/areas conducted serosurveys before 2009 (Table 2). By December 2017, HBsAg seroprevalence among children aged 5 years declined to <1% in 25 (69%) countries/areas based on national serosurveys (Table 2). Notably, China, which has the largest birth cohort in the region, was successful in decreasing the prevalence of HBV infection among children to <0.5%. In Laos, Papua New Guinea, Vietnam, and several of the Pacific Island countries, estimated HBsAg prevalence among children aged 5 years still exceeds 2%.

**TABLE 2:** Hepatitis B seroprevalence, by country/area — World Health Organization Western Pacific Region, 1976–2017

Country/Area	Year of most recent hepatitis B serosurvey	HBsAg prevalence (95% CI)	Year of verification of <1% HBsAg seroprevalence*
American Samoa	2011	0.2%	2014
Australia	2002	0.4% (0.0%–2.2%)	2012
Brunei	2011	0.1%	2013
Cambodia	2017	0.6% (0.3%–0.9%)^†^	NS^§^
China	2014	0.3% (0.2%–0.5%)	2012
Commonwealth of Northern Mariana Islands	2014	0.0% (0.0%–0.5%)	2017
Cook Island	2012	0.0%	2013
Federated States of Micronesia	2016	0.3% (0.1%–0.5%)	NS^§^
Fiji	2008^¶^	0.0%	NS
French Polynesia	2014	0% (0.0%–0.5%)	2016
Guam	2015	0.0%	2016
Hong Kong (China, SAR)	2009	0.8% (0.4%–1.2%)	2011
Japan	2010	0.2% (0.0%–0.4%)	NS
Kiribati	2014	3.3% (2.4%–4.6%)	NS
Laos	2012	1.7% (0.8%–2.6%)	NS
Macao (China, SAR)	2003	0% (0.0%–0.7%)	2008
Malaysia	2009	0.4% (0.2%–0.6%)	2011
Marshall Islands	2017	1.2% (0.6%–1.9%)	UR
Mongolia	2009	0.5% (0.4%–0.7%)	2012
Nauru	ND	―	NS
New Caledonia	ND	―	NS
New Zealand	2009	0.2% (0.0%–1.2%)	2012
Niue	2015	0.0%	2017
Palau	2008	0.0%	2013
Papua New Guinea	2012–2013	2.3%	NS
Philippines	2013	0.9%**	NS
Republic of Korea	2014	0.1%	2008
Samoa	2014	0.1%	NS
Singapore	2010	0.3% (0.1%–0.9%)	2015
Solomon Islands	2016	3.1% (2.0%–4.9%)^†^	NS
Tokelau	2014	0.0%	2016
Tonga	2005	0.8% (0.2%–2.5%)	2012
Tuvalu	1976	11.0%	NS
Vanuatu	1998	3.0%	NS
Vietnam	2011	2.2% (1.5%–3.1%)	NS
Wallis and Futuna	2012	0.9%	NS

**Abbreviations:** CI = confidence interval; HBsAg = hepatitis B surface antigen; ND = not done; NS = not submitted to the regional verification commission; SAR = special autonomous region; UR = under review by the regional verification commission.

* Verification is done by a regional commission of experts from the Hepatitis B Immunization Expert Resource Panel that determines if the country or area has reached the target of <1% HBsAg seroprevalence among children aged 5 years.

^†^ Preliminary data.

^§^ By December 2017, Cambodia and the Federated States of Micronesia had conducted nationally representative serosurveys and were subsequently verified as meeting the <1% HBsAg seroprevalence target in 2018.

^¶^ Fiji completed a subnational hepatitis B serosurvey in 2008 and is planning its first nationally representative survey for 2019.

** The Philippines conducted a nationally representative serosurvey in 2018 with preliminary results indicating a 0.7% HBsAg prevalence among children aged 5 years.

## Regional Verification of Hepatitis B Control Goal

In 2007, the WPR established the Hepatitis B Immunization Expert Resource Panel that independently advises on the status of and strategies for achieving the regional hepatitis B control goal.^§^ The Expert Resource Panel is an interdisciplinary team of 10–15 experts recognized in the field of hepatitis B, and members have expertise in immunization, epidemiology, virology, and hepatology. The Expert Resource Panel convenes verification panels to assess whether countries have met established regional control targets. The verification process includes review of data from nationally representative serosurveys conducted among children aged 5 years, who have already passed through the period of highest risk for perinatal and horizontal transmission of HBV. Panel members also review national and subnational HepB coverage data and supporting evidence of countries’ progress in monitoring and targeting high-risk populations including HBsAg-positive mothers and their exposed newborns. As of December 2017, 19 (53%) of the 36 countries/areas were verified by the Expert Resource Panel as having met the regional <1% prevalence target based on serosurvey and vaccination coverage data (Table 2).

## Progress Toward Elimination of Mother-to-Child Transmission of HBV

In 2017, the WPR established a goal for elimination of mother-to-child transmission (MTCT) of HBV by 2030, defined as achievement of a 90% reduction in new cases of chronic HBV infection, equivalent to 0.1% HBsAg seroprevalence among children aged 5 years, as part of the triple elimination framework. Key components of the WPR’s strategy to achieve elimination of MTCT of HBV include achieving ≥95% HepB-BD and HepB3 coverage, screening ≥95% of pregnant women for chronic HBV infection, administering hepatitis B immunoglobulin (HBIG) to infants born to HBsAg-positive mothers, and treating pregnant women eligible for treatment with antiviral drugs (*5*). As of December 2017, 19 (53%) of 36 countries/areas had developed national plans for viral hepatitis prevention; 26 (70%) countries/areas reported having established integrated routine antenatal screening programs for HIV and syphilis (data not shown); and 20 (56%) countries/areas had a national policy for routine antenatal HBsAg testing (Table 3). However, only two (6%) countries reported testing ≥95% pregnant women for HBsAg, and eight countries (22%) reported providing antivirals to infected mothers. In addition to providing timely HepB-BD and HepB3 vaccination for HBV-exposed infants, 10 (28%) countries/areas administered HBIG to newborns of HBsAg-positive mothers, and seven (19%) provided postvaccination serologic testing to determine the infection status of exposed infants.

**TABLE 3:** Policies and interventions implemented in elimination of mother-to-child transmission of hepatitis B ― World Health Organization (WHO) Western Pacific Region (36 countries/areas)* 2017

Policies/Interventions	No. (%) of areas		
	Implemented	Not implemented	Data not available
National plan for viral hepatitis^†^	19 (53)	6 (17)	11 (31)
National policy for antenatal HBsAg testing^†^	20 (56)	2 (6)	14 (39)
Maternal antivirals given for EMTCT of hepatitis B^†^	8 (22)	8 (22)	20 (56)
HBIG given to HBV-exposed infants^†^	10 (27)	6 (17)	20 (56)
Follow-up with PVST for HBV-exposed infants^†^	7 (19)	9 (25)	20 (56)
ANC1 ≥95%^§^	13 (36)	10 (28)	13 (36)
ANC4 ≥95%^§^	1 (3)	19 (53)	16 (44)
SBA present at ≥95% births^§^	17 (47)	10 (28)	9 (25)
Antenatal HBsAg testing coverage ≥95%^¶^	2 (6)	1 (3)	33 (92)
HB-BD coverage ≥95%**^,††^	15 (42)	17 (47)	2 (6)
HB3 coverage ≥95%**	18 (50)	16 (44)	2 (6)

**Abbreviations:** ANC1 = at least 1 antenatal care visit; ANC4 = at least 4 antenatal care visits; EMTCT = elimination of maternal-to-child transmission; HBsAg = hepatitis B surface antigen; HBIG = hepatitis B immunoglobulin; HBV = hepatitis B virus; HepB-BD = birth dose of monovalent hepatitis B vaccine; HepB3 = third dose of a hepatitis B containing vaccine; PVST = postvaccination serological testing; SBA = skilled birth attendant; UNICEF = United Nations Children’s Fund.

* The Pitcairn Islands is excluded from analysis because it does not report immunization coverage.

^†^ Information collected from countries in preparation for the Midterm review of the Regional Action Plan for Viral Hepatitis in the Western Pacific 2016–2020, December 13–14, 2018, Manila, Philippines.

^§^ UNICEF data. Monitoring the situation of children and women; https://data.unicef.org/ updated June 2018, data from 2006–2017.

^¶^ China, Wang et al. Bull World Health Organ 2015;93:52–6; Republic of Korea, unpublished study of the liver 2013; Mongolia, reported at the Informal Consultation on Validation of Elimination of Mother-to-child Transmission of HIV, Hepatitis B and Syphilis: Developing the Method for Validating Hepatitis B Elimination, February 27–28, 2018.

**WHO and UNICEF estimates for all 27 countries in the Western Pacific Region, unless otherwise specified; reported coverage for the remaining nine reporting areas and territories in the Western Pacific Region.

^††^ Japan and New Zealand are excluded for this indicator as these countries selectively offer hepatitis B birth dose to newborns of HBsAg-positive mothers or mothers with unknown HBsAg status.

## Discussion

During 2005–2017, the WPR achieved remarkable progress toward the regional hepatitis B control goal and elimination of MTCT of HBV. HepB has been introduced in all countries/areas; almost all countries/areas provide universal HepB-BD; coverage with HepB-BD and HepB3 increased by 35% and 22%, respectively; and 19 (53%) countries/areas were verified to have achieved the 2017 regional control target by December 2017. This success was corroborated by a 2016 disease modeling study that estimated regional prevalence to be 0.93% among children born in 2012.^¶^ This model also showed that immunization programs in the region prevented more than 37 million cases of chronic HBV infection, and averted more than seven million deaths related to hepatitis B that would have occurred in the lifetime of children born between 1990 and 2014 had hepatitis B vaccination programs not been established (*2*).

Interventions implemented to increase HepB-BD vaccination coverage included promoting community awareness about the need for HepB vaccination, especially the administration of a timely HepB-BD; building capacity and knowledge of health care staff to administer a timely birth dose; using HepB-BD outside the cold chain; and promoting institutional deliveries (*7*). WPR countries/areas have extensive experience using the highly heat stable monovalent HepB-BD outside the cold chain in areas that lack a reliable cold chain or have high home birth rates with limited health facility access. This use of the HepB-BD outside the commonly recommended storage temperatures of 35°F–46°F (2°C–8°C) for limited periods under monitored and controlled conditions, has been demonstrated to be safe and effective, with WHO suggesting outside-the-cold-chain use in settings where HepB-BD administration is restricted by access to cold storage (*1*). HepB-BD use outside the cold chain has been used to increase timely HepB-BD administration by 27% in Laos, 70% in China, and 150% in the Solomon Islands (*7*,*8*). Cambodia and China have national policies that encourage pregnant women to deliver in health facilities to reduce maternal and neonatal mortality by ensuring that mothers and newborns are examined by health care professionals within 24 hours of delivery. Institutional delivery also facilitates coordination of postnatal care services between maternal, neonatal, and child health programs and national immunization programs that can improve timely HepB-BD coverage (*7*,*9*).

To reach the global hepatitis B elimination goal of ≤0.1% HBsAg prevalence among children aged 5 years by 2030, WPR countries/areas need to achieve elimination of MTCT of HBV, because perinatal transmission accounts for a high proportion of chronic HBV infections among children (*1*). The triple elimination framework provides guidance for a coordinated delivery of services for immunization, HIV, sexually transmitted infections, and reproductive, maternal, neonatal, and child health to ensure that a timely HepB-BD is administered and high HepB3 coverage is achieved. The framework also provides guidance for implementation of complementary interventions in addition to vaccination to prevent perinatal HBV transmission, including routine testing of pregnant women and timely administration of hepatitis B immunoglobulin to exposed newborns. In addition, the framework suggests possible administration of antiviral drugs to mothers with high viral loads, while awaiting global guidance on its use to prevent MTCT of HBV (*5*).** In Cambodia, a modeling analysis indicated that offering an integrated package of services through the triple elimination platform could reduce MTCT of HBV by 76%, from 14.1% to 3.4%; syphilis by 51%, from 9.4% to 4.6%; and HIV by 8%, from 6.6% to 6.1%. It could prevent approximately 3,200 infant HBV infections annually at a cost of $114 USD per disability-adjusted life-year (*10*).

The WPR has significantly decreased the incidence of chronic HBV infection, with a few countries still requiring programmatic improvement in vaccination to achieve hepatitis B control. As the WPR expands implementation of interventions for elimination of MTCT of HBV, global and regional guidance is needed on 1) the use of monitoring indicators to assess the effect of these interventions on elimination of MTCT, 2) the appropriate frequency of costly serosurveys for verification of achievement of low HBsAg seroprevalence targets, and 3) the use of models to estimate infection prevalence from programmatic data to support countries in their control efforts and the elimination of MTCT verification process.

**Summary**

**What is already known about this topic?**

In 1990, chronic hepatitis B virus infection in the World Health Organization Western Pacific Region (WPR) was highly endemic (prevalence ≥8%).

**What is added by this report?**

During 2005–2017, regional hepatitis B vaccine birth dose (HepB-BD) and third dose (HepB3) coverage increased from 63% to 85% and from 76% to 93%, respectively. In 2017, 15 (42%) and 18 (50%) of 36 WPR countries/areas achieved ≥95% HepB-BD and HepB3 coverage, respectively. Chronic hepatitis infection in children declined to <1% in 25 (69%) countries/areas.

**What are the implications for public health practice?**

Continued commitment and enhanced coordination among programs that offer different hepatitis B prevention interventions are needed to achieve hepatitis B elimination by 2030.

## Footnotes

* The Western Pacific Region, one of the six regions of the World Health Organization, consists of 37 countries and areas with a total population of approximately 1.8 billion, including American Samoa (USA), Australia, Brunei, Cambodia, China, Commonwealth of the Northern Mariana Islands (USA), Cook Islands, Federated States of Micronesia, Fiji, French Polynesia (France), Guam (USA), Hong Kong (China), Japan, Kiribati, Laos, Macao (China), Malaysia, Marshall Islands, Mongolia, Nauru, New Caledonia (France), New Zealand, Niue, Palau, Papua New Guinea, Philippines, Pitcairn Islands (UK), Samoa, Singapore, Solomon Islands, Republic of Korea, Tokelau (New Zealand), Tonga, Tuvalu, Vanuatu, Vietnam, and Wallis and Futuna (France).

† The Pitcairn Islands, with a population of approximately 50 persons, does not have an immunization program and does not report immunization coverage data to WHO/UNICEF.

§ At the September 2018 Expert Resource Panel meeting (https://iris.wpro.who.int/bitstream/handle/10665.1/14321/RS-2018-GE-37b-PHL-eng.pdf), two new interim goals were proposed: 1) to reduce HBsAg prevalence to <1% among children aged 5 years in all countries and areas by 2025; and 2) in countries and areas that already have <1% HBsAg prevalence in children aged 5 years, to further reduce HBsAg prevalence to <0.3% by 2025. These are intended to be interim targets for countries to reach the hepatitis B elimination target of <0.1% HBsAg prevalence among children aged 5 years by 2030.

¶ Cambodia, Commonwealth of the Northern Mariana Islands, Federated States of Micronesia, French Polynesia, Guam, Niue, and Tokelau presented serosurvey evidence of reaching the <1% HBsAg prevalence target among children aged 5 years after 2016, suggesting that regional prevalence might have further declined.

** World Health Organization. Guidelines for the prevention, care, and treatment of persons with chronic hepatitis B infection. https://apps.who.int/iris/bitstream/handle/10665/154590/9789241549059_eng.pdf.
